# CD94 as a novel marker for immunophenotyping of leukemia and lymphoma in dogs

**DOI:** 10.3389/fvets.2025.1716800

**Published:** 2025-11-27

**Authors:** Anna Blockeel, Kristel Demeyere, Jonas Steenbrugge, Dominique Paepe, Adriana Krupa, Valeria Martini, Bert Devriendt, Evelyne Meyer

**Affiliations:** 1 Laboratory of Biochemistry, Department of Veterinary and Biosciences, Faculty of Veterinary Medicine, Ghent University, Ghent, Belgium; 2 Cancer Research Institute Ghent (CRIG) – Veterinary Oncology Network (VON), Ghent, Belgium; 3 Small Animal Clinic, Small Animal Department, Faculty of Veterinary Medicine, Ghent University, Ghent, Belgium; 4 Department of Veterinary Medicine and Animal Sciences, University of Milan, Lodi, Italy; 5 Laboratory of Immunology, Department of Translational Physiology, Infectiology and Public Health, Faculty of Veterinary Medicine, Ghent University, Ghent, Belgium

**Keywords:** dog, lymphoma, leukemia, natural killer, natural killer T, flow cytometry, immunophenotyping

## Abstract

CD94 is a natural killer (NK) cell receptor that also marks subsets of T cells, referred to as NKT cells. In humans, the role of CD94 as both an immune checkpoint and a potential therapeutic target has gained increasing attention. However, data about its expression in leukemia and lymphoma in dogs remain limited. This study aimed to explore CD94 expression in canine leukemia and nodal lymphoma, using a newly available anti-canine CD94 monoclonal antibody in a multicolor flow cytometry panel. Surplus blood and lymph node aspirate samples from eleven client-owned dogs (leukemia: *n* = 7, lymphoma *n* = 4) and two clinically healthy controls, were analyzed. The control dogs as well as most cases showed low CD94^+^ lymphocyte frequencies, consistent with a non-neoplastic population. However, markedly expanded CD94^+^ populations were identified in two out of four of the T cell chronic lymphocytic leukemia (T-CLL) cases. In one of them, the neoplastic population was uniformly CD3^+^CD8^+^CD94^+^, while the other showed a heterogeneous mixture of CD3^+^CD8^+^CD94^+^ and CD3^−^CD8^+^CD94^+^ lymphocytes. Our findings demonstrate that the canine-specific CD94 antibody can be applied to both blood and lymph node samples in a diagnostic flow cytometry setting. While CD94 expression was infrequent overall, its detection in a subset of T-CLL cases highlights the need for larger studies to determine its diagnostic and therapeutic value in canine leukemia and lymphoma.

## 1 Introduction

CD94, or the killer cell lectin like receptor, subfamily D, member 1 (KLRD-1), belongs to the C-type lectin family of proteins and recognizes non-classical major histocompatibility complex class 1 (MHC-I) molecules (1, 2). In humans, CD94 is expressed by NK cells as well as natural killer T (NKT) cells, the latter being a T cell subset that shares features of both NK and T cells. CD94 heterodimerizes with members of the natural killer group 2 (NKG2) family, resulting in either an activating or inhibitory effect on NK(T) cell function (3, 4). Anti-human CD94 antibodies were shown to cross react with canine leukocytes, revealing an expression pattern on their surface comparable to human leukocytes (5). This led to the development of an anti-canine CD94 monoclonal antibody (mAb), which recently became commercially available and was shown to bind to both canine NK and NKT cells, similar to their human counterparts (6).

NK cells are able to kill without prior sensitization and are especially known to target virus-infected and cancer cells, both by recognizing ligands on the target cell surface directly and through antibody-dependent cellular cytotoxicity (ADCC) (7–10). NKT cells can be divided into two subsets that differ in phenotype and function: invariant NKT (iNKT) and NKT-like cells. iNKT cells express an invariable T cell receptor (TCR), recognizing glycolipids presented by the MHC-like molecule CD1d, while conventional T cells expressing NK receptors are described as NKT-like cells (11, 12).

However, the precise function and regulation of NK(T) cells remains not fully understood, particularly in dogs. Efforts to improve their characterization and explore their potential as immunotherapeutic strategies for cancer are ongoing (12–15). Given the role of NK cells in cancer cell killing and the dual role of CD94 (both inhibitory and activating), CD94 has emerged as a therapeutic target in humans, both within the tumor immune microenvironment as well as directly targeting tumors of cytotoxic cell origin (16–18). However, knowledge of CD94 expression in the tumor immune microenvironment and on tumor cells is essential in order to evaluate the potential of these therapeutics. The inhibitory CD94-NKG2A heterodimer has emerged as a new kid on the block of immune checkpoints, since its expression on human CD8^+^ T cells has been described in the immune microenvironment of multiple solid cancers (19). By contrast, expression of CD94 on the surface of the tumor cell itself in human NK and/or T cell malignancies is less well described. If these studies are limited in humans, they are currently non-existent in dogs. Additionally, to the authors' knowledge, the commercially available canine mAb has not yet been applied to clinical samples, especially to lymph node fine needle aspirates (LNAs).

This study aimed to provide a preliminary exploration of CD94 expression in canine leukemia and lymphoma, both within the tumor immune microenvironment and on tumor cells, by applying the novel canine CD94 mAb in a multicolor flow cytometric (FCM) panel with CD3 and CD8 to clinical samples from client-owned dogs. Supported by recent single cell transcriptomics data from dogs, CD3^−^CD8^+/−^CD94^+^ cells were defined as NK cells, while CD3^+^CD8^+/−^CD94^+^ cells were defined as NKT-like cells (20, 21). As new therapeutic strategies are rapidly emerging in veterinary oncology, accurate immunophenotyping of these cancers will be crucial to allow careful canine patient selection in the era of personalized medicine.

## 2 Materials and methods

### 2.1 Sample selection and classification

Surplus canine peripheral blood and LNA samples were collected through the clinical FCM diagnostic service of the Laboratory of Biochemistry (Faculty of Veterinary Medicine, Ghent University, Ghent, Belgium) between February 2024 and August 2025. The samples originated from client-owned dogs presented at the faculty's Small Animal Clinic and at private veterinary practices for leukemia (blood samples) or lymphoma (LNA samples). Inclusion criteria were determined as follows: >2 x 10^6^ leukocytes available for CD94 staining, sample viability >50% and access to the dog's medical file. Prior corticosteroid use was noted, but samples were not excluded. Samples with a B cell immunophenotype were not considered. Clinically healthy dog samples were obtained from client-owned dogs with informed owner consent under Ethical Committee approval (reference number EC2023-002).

Medical files of the included dogs were obtained from referring veterinarians and thoroughly reviewed. Samples were classified into leukemia or lymphoma subtypes based on the dog's history, physical exam, blood examination (hematology and serum biochemistry), (immuno)cytology and PCR for antigen receptor rearrangement (PARR), depending on availability. Histopathology was not performed for any of the dogs enrolled in the study. FCM diagnostic panels, evaluated by a single experienced operator (VM), were used for classification in all neoplastic samples.

### 2.2 Flow cytometry

Blood samples were collected in ethylenediaminetetraacetic acid (EDTA)-coated tubes, while LNA samples were collected in Dulbecco's modified eagle medium (DMEM)-containing tubes. First, diagnostic antibody panels (CD45, CD34, CD21, CD3, CD5, CD4, CD8, CD14, and MHCII) were applied. Second, our CD94 antibody panel consisting of CD3, CD8, and CD94 was applied to the residual sample. An overview of all antibody characteristics can be found in Supplementary Table S1.

**Figure 1:**
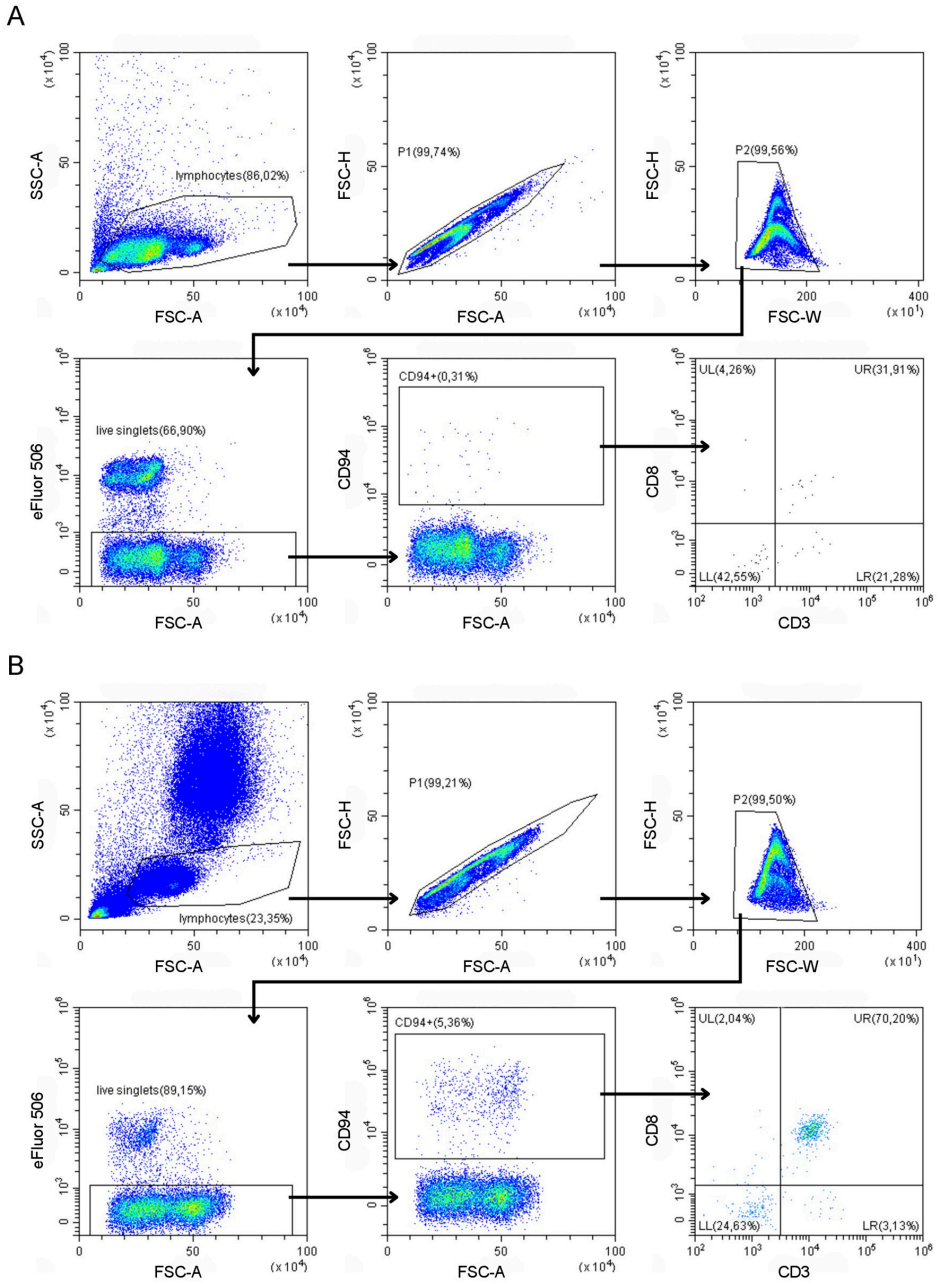
Gating strategy of the CD94-panel (containing CD3, CD8, and CD94 antibodies) in a representative lymph node fine needle aspirate **(A)** and peripheral blood **(B)** sample from a clinically healthy dog.

After erythrocyte removal using a lysing buffer (90% NH_4_Cl, 10% KHCO_3_ and 0,37% EDTA), cells were washed in phosphate buffered saline (PBS), counted and up to 1 x 10^6^ cells/well were placed in a 96-well conical bottom microtiter plate. Cells were stained with a viability dye (Fixable Viability Dye eFluor 506, Invitrogen, Waltham, MA, USA) at a 1/1,000 dilution in PBS for 30 min at 4 °C. Subsequently cells were washed and incubated with an Fc-blocking buffer [fetal bovine serum (FBS) diluted 1/10 in PBS] for 10 min at room temperature. Cells were washed again and incubated with the corresponding antibodies in 100 μl PBS for 30 min at 4°C at appropriate concentrations as determined following in-house titration (Supplementary Table S1). Cells were washed a final time, resuspended in PBS and acquired using a 3-laser CytoFLEX (Beckman Coulter, Brea, CA, USA) at the Core Flowcytometry at Ghent University (Ghent, Belgium). Appropriate compensation was applied to account for fluorochrome spill-over. Unstained controls were included for all samples to determine autofluorescence. Fluorescence minus one (FMO) and isotype controls of CD94 (mouse IgG1-Alexa Fluor^®^647, Bio-Rad, Hercules, CA, USA) were included with randomly selected samples to ensure staining specificity (Supplementary Figure S2). All samples were analyzed using CytExpert FCM analysis software. The gating strategy of our optimized CD94-panel in both blood and LNA is shown in Figure 1. Number of events was recorded within the CD94^+^ gate and if >150, CD3 and CD8 expression within this gate was additionally evaluated.

## 3 Results

### 3.1 Classification of samples

Eleven leukemia or lymphoma dogs (blood or LNA samples, respectively) and two healthy dogs (for which both blood and LNA were collected) were included for a total of 15 samples. An overview of clinical characteristics, including breed, age, sex, and weight, can be found in Table 1. Three dogs presented with a marked leukocytosis (range: 27,130–514,620 leukocytes/μL), no or moderate peripheral lymphadenopathy and an expanded population of intermediate to large CD34^+^ cells on blood FCM. In the absence of definitive lineage marker characterization allowing differentiation between acute myeloid and lymphoblastic leukemia, these samples were classified as acute leukemia (AL). One AL dog received corticosteroid therapy prior to sampling. Four dogs presented with leukocytosis (range: 81,000–179,600 leukocytes/μL), no or moderate peripheral lymphadenopathy and an expanded population of small to intermediate CD3^+^CD5^+^CD8^+^MHCII^+^ lymphocytes on blood FCM, compatible with T cell chronic lymphocytic leukemia (T-CLL). One T-CLL dog received corticosteroid therapy prior to sampling. Three dogs presented with peripheral lymphadenopathy and an expanded population of intermediate to large CD3^+^CD5^−^CD4^+^CD8^−^ (*n* = 2) or CD3^−^CD4^−^CD8^+^ (*n* = 1) cells on LNA FCM and were subsequently classified as nodal T cell lymphoma—not otherwise specified (NTCL-NOS). Finally, one dog presented with mild lymphadenopathy and an expanded population of small to intermediate CD45^−^CD5^+^CD21^+^ lymphocytes on LNA FCM, compatible with T zone lymphoma (TZL). Two clinically healthy dogs, for which both blood and LNA samples were included to assess physiological CD94 expression patterns, had no abnormalities on physical exam or routine hematology/serum biochemistry.

**Table 1:** Overview of the clinical characteristics for all dogs.

**Classification**	**Breed**	**Age (years)**	**Sex**	**Weight (kg)**	**Sample matrix**	**FCM (lymphocyte gate)**								
						**CD45**	**CD34**	**CD21**	**CD3**	**CD5**	**CD4**	**CD8**	**CD14**	**MHCII**
AL	Border Collie	11.94	FS	15.2	Blood	97%	86%	<1%	9%	2%	1%	<1%	0%	4%
AL	Golden Retriever	9	NA	29.3	Blood	100%	95%	<1%	1%	<1%	NA	<1%	<1%	NA
^**cs**^AL	Mixed Breed	6.13	FS	37.4	Blood	99%	64%	3%	6%	<1%	5%	3%	<1%	8%
^**cs**^T-CLL	Labradoodle	6.61	FS	14.3	Blood	99%	<1%	4%	97%	80%	<1%	98%	<1%	99%
T-CLL	NA	NA	NA	NA	Blood	98%	<1%	3%	91%	90%	5%	96%	2%	94%
^*^T-CLL	American Staffordshire Terrier	8.75	NA	17.4	Blood	100%	3%	<1%	98%	96%	1%	96%	<1%	99%
^**^T-CLL	French Bulldog	11.82	MC	21.6	Blood	100%	1%	1%	74%	98%	<1%	97%	0%	98%
NTCL-NOS	Bernese Mountain Dog	6.42	M	54.25	LNA	NA	1%	7%	92%	NA	82%	<1%	NA	NA
NTCL-NOS	Hovawart	10.98	M	40	LNA	98%	<1%	<1%	98%	1%	92%	<1%	<1%	NA
NTCL-NOS	Whippet	11.46	M	NA	LNA	NA	6%	21%	14%	NA	14%	69%	NA	NA
TZL	American Staffordshire Terrier	8.87	MC	28	LNA	27%	<1%	86%	85%	85%	15%	76%	<1%	99%
Healthy	Bernese Mountain Dog	4.23	F	37.8	Blood	NA	2%	10%	57%	58%	30%	16%	NA	NA
					LNA	NA	6%	41%	61%	53%	31%	17%	NA	NA
Healthy	Beagle	2.21	F	14.5	Blood	NA	1%	9%	84%	NA	NA	19%	NA	NA
					LNA	NA	1%	19%	82%	NA	61%	13%	NA	NA

Dogs with 96% (^*^) and 24% (^**^) CD94^+^ lymphocyte frequencies are marked.

FCM, flow cytometry; AL, acute leukemia; T-CLL, T cell chronic lymphocytic leukemia; NTCL-NOS, nodal T cell lymphoma - not otherwise specified; TZL, T-zone lymphoma; LNA, lymph node fine needle aspirate; M, male; MC, male castrated; F, female; FS, female spayed; cs, prior use of corticosteroids; NA, not available.

### 3.2 CD94 expression in blood and LNA samples of dogs with leukemia or lymphoma

**Figure 2:**
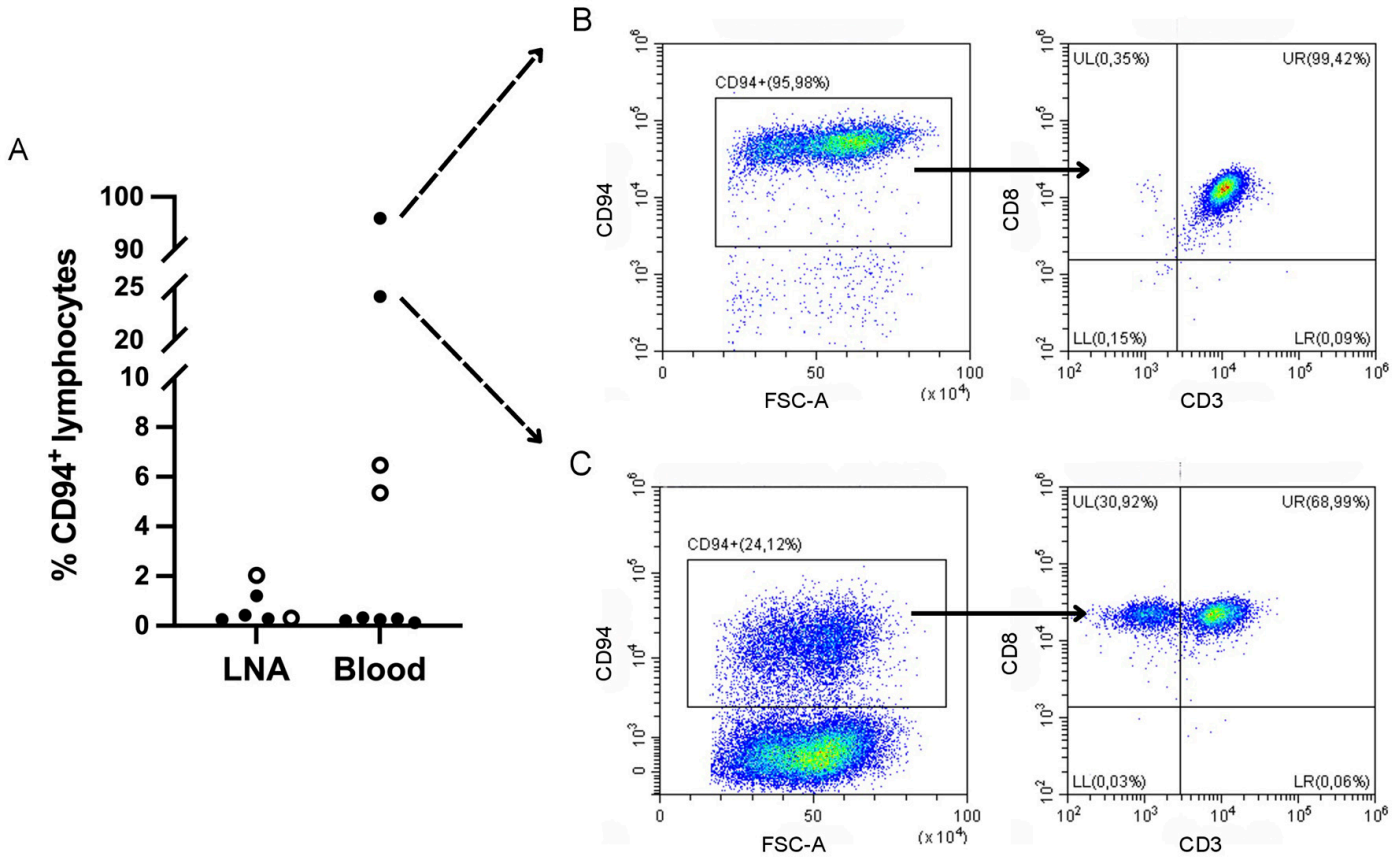
CD3^−^CD8^−^CD94^+^ natural killer and CD3^+^CD8^+^CD94^+^ natural killer T (NKT) cell frequencies in dogs with variable hematopoietic malignancies and clinically healthy dogs. **(A)** Distribution of CD94^+^ lymphocyte frequencies in all dogs. Data from clinically healthy dogs is represented by a circle symbol. Frequencies are low in most dogs, except for two dogs diagnosed with T cell chronic lymphocytic leukemia (T-CLL). LNA, lymph node fine needle aspirate. **(B)** Peripheral blood sample from a dog with T-CLL and the highest CD94 frequencies, showing CD3 and CD8 expression on almost all CD94^+^ lymphocytes, classifying them as NKT cells. **(C)** Peripheral blood sample from a dog with T-CLL and intermediate CD94 frequencies. CD3 and CD8 expression is seen on a majority of CD94^+^ lymphocytes, classifying them as NKT cells, although a subpopulation of CD3^−^CD8^+^, classified as NK cells, is present.

**Figure 3:**
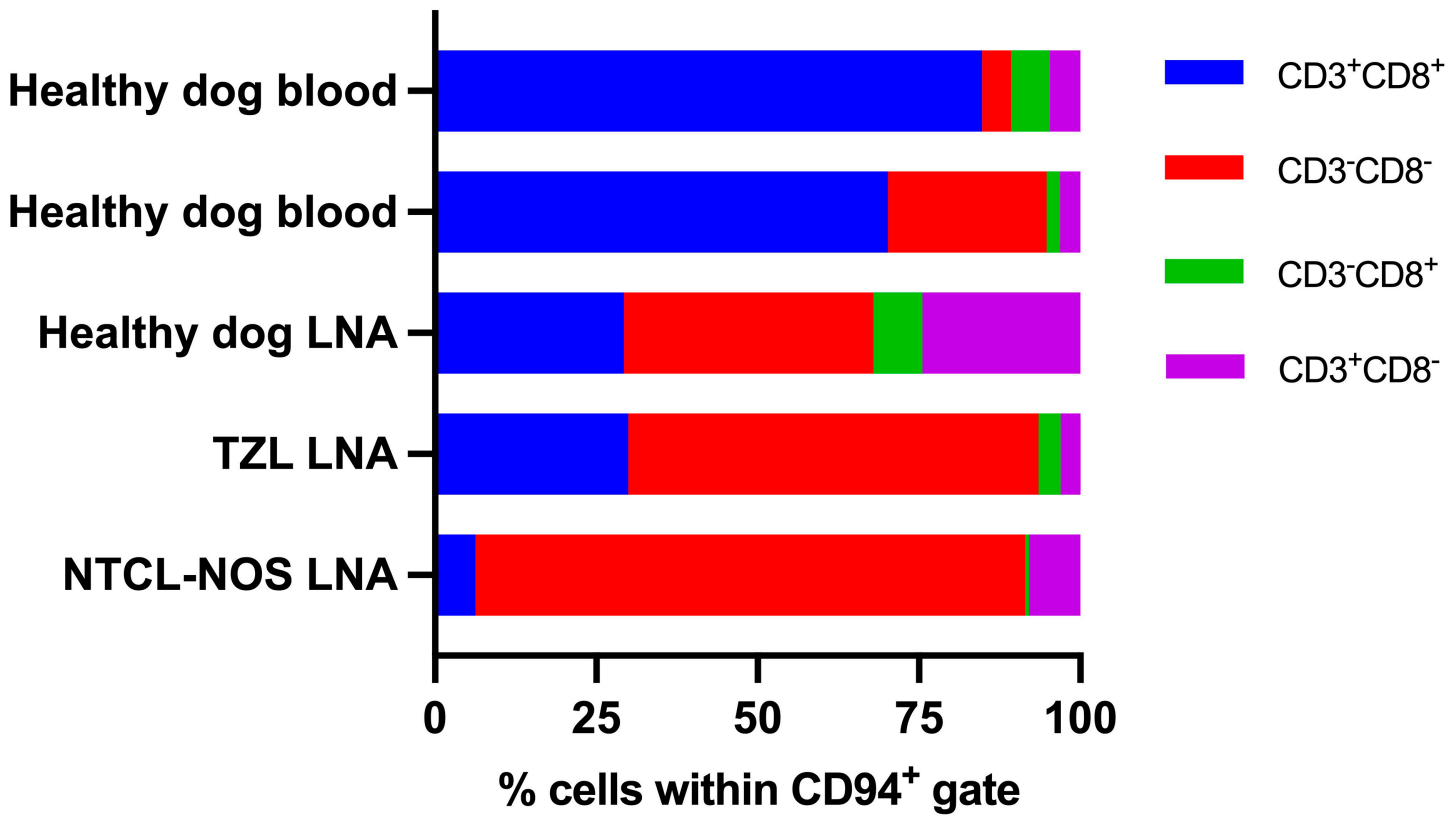
CD3 and CD8 expression within the CD94^+^ lymphocyte population of samples with low CD94 frequencies. Samples with <150 CD94^+^ events were not evaluated. A CD3^+^CD8^+^ natural killer T-like immunophenotype predominates in blood samples, while a CD3^−^CD8^−^ natural killer immunophenotype predominates in lymph node aspirates (LNAs). In LNAs, this natural killer immunophenotype becomes more prominent with more aggressive disease. TZL, T zone lymphoma; NTCL-NOS, nodal T cell lymphoma – not otherwise specified.

To explore CD94 expression in canine leukemia and lymphoma, all samples were analyzed using our optimized CD94-panel and gating strategy (Figure 1). CD94^+^ lymphocyte frequencies were generally low in both blood and LNA samples of diseased and clinically healthy dogs (Figure 2). Sufficient CD94^+^ events were recorded in 7/15 samples, allowing further evaluation of CD3 and CD8 expression within this gate to immunophenotype these CD94^+^ cells. Two dogs showed higher CD94^+^ lymphocyte frequencies than the other included dogs and both were diagnosed with T-CLL. One dog displayed a very high CD94^+^ frequency of 96% in blood. Of these CD94^+^ lymphocytes, 99% were positive for the lineage markers CD3 and CD8, classifying them as NKT-like cells. The second dog had an intermediate CD94^+^ lymphocyte frequency of 24% in blood. While 69% of these lymphocytes were positive for the lineage markers CD3 and CD8, classifying them as NKT-like cells, 31% were CD3^−^CD8^+^, classifying them as NK cells (Figure 2). Neither of these dogs received corticosteroid therapy prior to sampling (Table 1). In contrast to these two dogs with T-CLL and higher CD94 frequencies, frequencies were low in the other two T-CLL dogs, in other malignancy types or in clinically healthy dogs. A CD3^+^CD8^+^ NKT-like immunophenotype predominated the CD94^+^ lymphocyte population in blood of two healthy dogs, while a CD3^−^CD8^−^ NK immunophenotype predominated the CD94^+^ lymphocyte population in the LNA samples of one healthy, one TZL and one NTCL-NOS dog. NK immunophenotype frequencies were highest in the CD94^+^ lymphocytes of the NTCL-NOS sample, followed by the TZL sample and were lowest in the healthy LNA (Figure 3).

## 4 Discussion

Our work shows that the anti-canine CD94 monoclonal antibody can readily be applied in a multicolor FCM panel on clinical samples of both canine blood and LNAs. Prior studies have worked with peripheral blood mononuclear cells of laboratory dogs (6, 22, 23). As such, the use of clinical samples and especially LNA samples has not been previously reported. We have shown that, even in whole blood/LNAs, this antibody provided a sufficiently clean staining in both matrices and is therefore a qualitative option for clinical FCM.

Overall, CD94^+^ lymphocytes were infrequent in both diseased and healthy dogs. Where a sufficient number of events allowed further immunophenotyping of the CD94^+^ cells, they appeared to be skewed toward a CD3^+^CD8^+^ NKT-like immunophenotype in the blood of two healthy dogs, while in LNAs of one healthy, one TZL and one NTCL-NOS dog, they appeared to be skewed toward a CD3^−^CD8^−^ NK cell immunophenotype. Among the LNAs, the CD3^−^CD8^−^ NK cell immunophenotype of CD94^+^ cells was least frequent in the healthy and highest in the NTCL-NOS sample, with the TZL falling in between. NTCL-NOS is considered a clinically aggressive tumor, while TZL is typically indolent. Thus, the NK cell immunophenotype of CD94^+^ cells may become more prominent with more aggressive disease. As the low frequencies of CD94^+^ cells in these samples likely reflect non-neoplastic cells, their characterization might help to elucidate the tumor immune microenvironment of these tumors.

Additionally, we have shown that an expanded population of CD94^+^ lymphocytes is present in two out of four T-CLL samples. In one dog, almost all lymphocytes were consistently CD3^+^CD8^+^CD94^+^, reflecting a dominant, uniform neoplastic population. In the other dog however, CD94^+^ lymphocyte frequencies were lower and those CD94^+^ cells represented both a CD3^+^CD8^+^ as well as a CD3^−^CD8^+^ subset. In this dog, the dominant, neoplastic population consists of CD3^+^CD5^+^CD8^+^MHCII^+^ cells (Table 1). The CD94^+^ cells may therefore reflect aberrant expression of CD94 and loss of CD3 on the tumor cells. Alternatively, they may reflect a large reactive, non-neoplastic population of NKT and NK cells. To distinguish between a heterogenous neoplasm and a mixed reactive/neoplastic population, PARR could be considered.

Although the human classification of T and NK malignancies cannot entirely be extrapolated to their canine counterparts, some parallels can be drawn. Canine CD8^+^ T-CLL appears to correspond best to human CD8^+^ large granular lymphocytic leukemia (LGLL), a mostly indolent disease common in older people. One study using FCM of five CD8^+^ LGLL patients described CD94^+^ lymphocytes in blood samples of all patients, ranging from 30%−90% of all CD8^+^ T cells (24). A more recent study describing 43 LGLL samples, most of them CD8^+^, classified 56% as CD94 positive, with a median percentage of 30% positive cells (25). These findings are in line with our observations in canine T-CLL. Considering AL, comparisons are less straightforward since this term encompasses both acute myeloid and acute lymphoblastic leukemia, with several other subtypes described within those groups in humans. Finally, nodal NK and T cell lymphomas are a heterogenous group in both humans and dogs. Overall, CD94 positivity appears to be infrequent across human forms, except in cases with clear NK-lineage (25–27). Although tumors with proven NK-lineages appear to be universally positive, they are also extremely rare (28). Similarly, we did not identify a CD3^−^CD94^+^ NK-lineage in any of our canine lymphoma samples. However, although CD94 is expressed on the majority of canine NK cells, using additional NK cell markers such as CD5^dim^ or NKp46 might allow identification of a CD94^−^ NK cell lineage in these samples (6, 14).

The main limitation of this study is its small sample size, in part due to the rarity of confirmed NK cell neoplasia and the lower frequency of T cell compared to B cell leukemia or lymphoma in dogs. First of all, a larger number of clinically healthy dogs is needed to determine normal ranges of CD94^+^ cells and their immunophenotype in both blood and lymph nodes. Secondly, more samples of non-CD94^+^ tumors are needed to determine the immunophenotype and function of non-neoplastic CD94^+^ cells in the tumor immune microenvironment. Lastly, although we did not observe CD94-positive nodal lymphomas or ALs, a larger sample size potentially might allow this. As a minor limitation, consideration should be given to the fact that corticosteroid therapy in one AL and one T-CLL dog might have influenced expression patterns in those dogs.

Future studies should focus not only on increasing sample size, but also on elucidating the prognostic role of CD94. Given its dual role (both inhibitory and activating), CD94 could have either pro- or anti-tumor effects in the tumor immune microenvironment as well as on tumor cells. Prospective studies collecting survival data may shed light on this.

In conclusion, the commercial canine-specific CD94 mAb can be used to evaluate CD94 expression patterns in blood and LNAs of healthy and leukemia or lymphoma dogs. This may aid in elucidating the immunophenotype and function of non-neoplastic CD94^+^ cells in the tumor immune microenvironment. Additionally, it can be used to identify CD94^+^ cases of known tumor types, such as T-CLL. Further research is needed to better assess the prevalence of CD94^+^ CLLs and possibly other lymphoid neoplasms and determine their clinical significance, potentially opening up new therapeutic possibilities tailored to the individual canine patient.

## Data Availability

The raw data supporting the conclusions of this article will be made available by the authors, without undue reservation.
