# Supplementary material for: CD94 as a novel marker for immunophenotyping of leukemia and lymphoma in dogs
Source: Front Vet Sci. 2025 Nov 27;12:1716800. doi: 10.3389/fvets.2025.1716800 (PMC12696703; doi:10.3389/fvets.2025.1716800)
Supplement: Supplementary file 1 [file Table_1.pdf]

Supplementary Table S1: Overview of the antibodies used in this study.

**Antigen**	**Clone**	**Fluorochrome**	**Host**	**Isotype**	**Dilution (in 100µL)**	**Supplier**
CD3	CA17.2A12	FITC	Mouse	IgG1	1/10	Bio-Rad
CD4	YKIX 302.9	R-PE	Rat	IgG2a	1/10	Bio-Rad
CD5	YKIX322.3	Pacific Blue	Rat	IgG2a	1/50	Bio-Rad
CD8	YCATE55.9	PerCP-eFluor™ 710	Rat	IgG1	1/20	Thermo Fisher Scientific
CD14	TÜK4	R-PE	Mouse	IgG2a	1/10	Bio-Rad
CD21	CA2.1D6	Alexa Fluor® 488	Mouse	IgG1	1/50	Bio-Rad
CD21	CA2.1D6	R-PE	Mouse	IgG1	1/10	Bio-Rad
CD34	1H6	Alexa Fluor® 647	Mouse	IgG1	1/10	Bio-Rad
CD34	1H6	PE	Mouse	IgG1	1/20	Thermo Fisher Scientific
CD45	YKIX716.13	Alexa Fluor® 647	Rat	IgG2b	1/25	Bio-Rad
CD94	8H10	Alexa Fluor® 647	Mouse	IgG1	1/10	Bio-Rad
MHCII	YKIX334.2	Alexa Fluor® 647	Rat	IgG2a	1/50	Bio-Rad
