# Supplementary material for: CD94 as a novel marker for immunophenotyping of leukemia and lymphoma in dogs
Source: Front Vet Sci. 2025 Nov 27;12:1716800. doi: 10.3389/fvets.2025.1716800 (PMC12696703; doi:10.3389/fvets.2025.1716800)
Supplement: Supplementary file 2 [file Image_1.pdf]

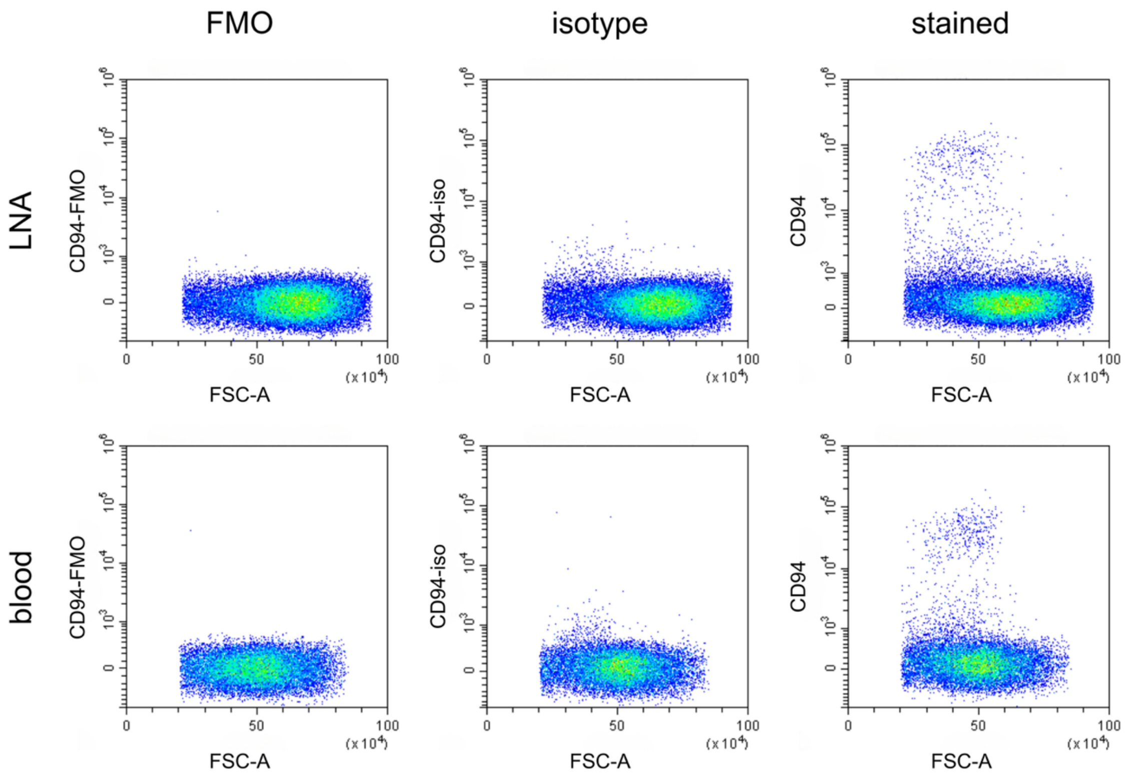


**Supplementary Figure S2:** Representative dot plots of fluorescence minus one (FMO) and isotype controls for CD94, shown on a lymph node fine needle aspirate (LNA) and peripheral blood sample of a dog diagnosed with B cell lymphoma (data not shown). Isotype controls show a small amount of low intensity background staining, which is easily discernible from higher intensity specific CD94 staining.
